# Evaluation of Mediterranean Agricultural Residues as a Potential Feedstock for the Production of Biogas via Anaerobic Fermentation

**DOI:** 10.1155/2015/171635

**Published:** 2015-11-02

**Authors:** Christos Nitsos, Leonidas Matsakas, Kostas Triantafyllidis, Ulrika Rova, Paul Christakopoulos

**Affiliations:** ^1^Laboratory of General and Inorganic Chemical Technology, Department of Chemistry, Aristotle University of Thessaloniki, 54124 Thessaloniki, Greece; ^2^Biochemical Process Engineering, Division of Chemical Engineering, Department of Civil, Environmental and Natural Resources Engineering, Luleå University of Technology, 971-87 Luleå, Sweden; ^3^Chemical Process and Energy Resources Institute, Centre for Research and Technology-Hellas (CPERI/CERTH), 57001 Thessaloniki, Greece

## Abstract

Hydrothermal, dilute acid, and steam explosion pretreatment methods, were evaluated for their efficiency to improve the methane production yield of three Mediterranean agricultural lignocellulosic residues such as olive tree pruning, grapevine pruning, and almond shells. Hydrothermal and dilute acid pretreatments provided low to moderate increase in the digestibility of the biomass samples, whereas steam explosion enabled the highest methane yields to be achieved for almond shells at 232.2 ± 13.0 mL CH_4_/gVS and olive pruning at 315.4 ± 0.0 mL CH_4_/gVS. Introduction of an enzymatic prehydrolysis step moderately improved methane yields for hydrothermal and dilute acid pretreated samples but not for the steam exploded ones.

## 1. Introduction

The replacement of conventional fossil fuels with alternative sources is dictated by a number of concerns, regarding future sustainability of feedstock, energy security, reduction of greenhouse gas emissions, and support of local economy. To meet these significant challenges extensive research for different feedstocks and energy production processes is currently underway. To this end, solid, liquid, and gaseous biofuels that are produced from renewable sources and waste streams of biological origin are very promising. Bioethanol [1], biohydrogen [2], and biogas [3] are amongst the most widely researched types of biofuels.

First generation biodiesel (using high quality edible vegetable oils as feedstock) and bioethanol (using edible sugar raw materials) are already in commercial use in the transport sector but have also received a lot of criticism due to “food versus fuel” issue. During the last years, the second generation biodiesel and bioethanol originating from waste/low quality vegetable oils and lipids or cellulosic raw materials, respectively, have gained increased research interest in an effort to replace their first generation analogues. The anaerobic digestion process used for the production of biogas, on the other hand, possesses several advantages compared to the typical bioethanol and biodiesel processes [4]. These include the ability to convert a wide variety of feedstocks with widely different compositions into biogas by applying the same basic process, the elimination of sterile requirements due to the microbial diversity of the digesters, the spontaneous separation of the gaseous product from the liquid medium, and full utilization of the remaining solid digestate and liquid effluent as fertilizers. The high volumetric methane content of the biogas promises an equally high energy yield.

Methane production via anaerobic digestion is a process consisting of four main stages, hydrolysis, acidogenesis, acetogenesis/dehydrogenation, and methanation, which are carried out by different consortia of anaerobic microorganisms [5]. In the first hydrolytic stage, complex polymers such as carbohydrates, proteins, and lipids are converted into soluble oligomers and monomers. During acidogenesis these are converted to volatile fatty acids and then into acetate and hydrogen before being finally converted to methane. This complex process allows for the lack of aseptic conditions during fermentation as well as the utilization of such diverse feedstock as animal manure [6], wastewater sludge [7], and food waste [8] as well as starch and lignocellulosic energy crops [4].

Utilization of lignocellulosic wastes such as agricultural and forestry residues, as well as byproducts of the related industries, is considered promising feedstock for the production of biofuels due to their renewable nature, abundance, and the possibility to support the local economies. Within this scope, agricultural residues from olive tree pruning, grapevine pruning, and almond shells are of great importance for Southern Europe and Mediterranean Basin countries. As an example, Spain, Italy, and Greece account for around 70% of the world olive oil production [9], while the production of wine and various nuts also represents a major agricultural activity in Mediterranean countries. The great amount of byproducts from these activities, such as pruning from olive tree and grapevine and shells and hull from nuts, has not yet been incorporated into an efficient utilization process scheme. Rather, pruning is either burned or scattered in the field as fertilizer and shells are burned for the production of energy in the food industry.

The aim of the current work was to evaluate the potential of these three lignocellulosic agricultural residues as potential feedstock for the production of biogas through anaerobic digestion. For this purpose three different pretreatment methods, that is, hydrothermal, dilute acid, and steam explosion, as well as enzymatic hydrolysis of the pretreated solids was applied and the effect of all the pretreatment and enzymatic hydrolysis processes on the biomethane yield was studied.

## 2. Materials and Methods

### 2.1. Feedstock and Inoculum

Pruning from olive tree and grapevine as well as almond shells from the Northern Greece region of Chalkidiki was used for the current work. The pruning contained only the bigger branches, separated from small twigs and leaves. The inner hard almond shells that were used were also separated from the outer soft hulls. After air-drying in the laboratory the biomass samples were passed through a knife mill with a 1 mm sieve for size reduction. The volatile solids (VS) and total solids (TS) content of the initial biomass samples as well as the inoculum used can be seen in Table 1.

During the digestions, a thermophilic anaerobic sludge was used as inoculum. The sludge was collected from a biogas producing plant located in Boden, Sweden, where food wastes are codigested with sewage sludge at 55°C.

### 2.2. Pretreatment and Enzymatic Hydrolysis

The three available biomass types were subjected to three types of pretreatment, hydrothermal pretreatment (HT), dilute acid pretreatment (DA), and steam explosion (SE). Due to the limited number of publications on the use of these materials as feedstock for the production of biofuels, the pretreatment conditions used in this study were the same as those used for olive pruning [10, 11] and can be seen in Table 2.

The pretreated biomass samples were also enzymatically hydrolyzed with the commercial enzyme preparation Cellic CTec2 from Novozymes (Bagsærd, Denmark). Two different approaches were utilized for the enzymatic hydrolysis of the pretreated biomass. In the one-step approach the enzymes were added directly to the sludge in order to achieve an enzyme activity equal to 15 FPU/g biomass, while in the two-step approach the biomass was saccharified prior to the addition to the sludge. In the latter case the saccharification was performed at 50°C for 8 hours and 23% w/w DM. For both approaches the enzyme loading was 15 FPU/g biomass.

### 2.3. Analytical Methods

Total solids (TS) and volatile solids (VS) contents were determined as previously described [12]. The enzyme activity of Cellic CTec2 was measured according to the standard protocol [13] and was 240 FPU/mL.

### 2.4. Biochemical Methane Potential (BMP)

The BMP tests were performed as previously described [12] using the Automatic Methane Potential Test System II (AMPTS II, Bioprocess Control AB, Lund, Sweden). Digestion took place at 55°C and the I/S (inoculum to substrate in terms of VS) was adjusted to 2. Incubation was performed in 500 mL glass bottles filled with a total of 400 g of sludge and substrate. Slow mixing (10 min mixing and 1 min rest) of the sludge was performed with the help of motor fitted at the top of each flask. Each flask was connected to a 100 mL flask containing 80 mL of 3 M NaOH in order to trap CO_2_ and thymolphthalein as pH indicator. Finally, methane volume was measured at the flow meter unit. In each batch of digestion, two controls, namely, only sludge and sludge with the enzymes (if applicable), were also included in order to calculate the methane production from the organic load present in the sludge and the enzyme digestion, respectively. These amounts of methane were finally retracted from the methane production in order to calculate the pure methane production from the substrate only. Finally, in order to evaluate the quality of the sludge, a positive control was also included where microcrystalline cellulose (Avicel) was used as substrate. It is worth mentioning that the digestion was terminated when no significant amounts of methane were detected.

## 3. Results and Discussion

During this work we evaluated three different agricultural residues, namely, vine and olive pruning and almond shells, as a potential feedstock for the production of biogas via anaerobic digestion. As discussed previously, the chosen agricultural species could be of high economic interest in countries in the Mediterranean basin, due to the high amount of lignocellulosic residues that are currently underutilized and whose potential to be converted into high-value biofuels has hardly been explored.

The three lignocellulosic feedstocks were evaluated for their biomethane potential in their initial untreated form and after pretreatment using three different methods, hydrothermal (HT), dilute acid (DA), and steam explosion (SE). Furthermore the solids derived from the three pretreatment methods also underwent a further enzymatic hydrolysis both prior to and during anaerobic digestion.

The volatile solids (VS) and total solids (TS) content of the untreated as well as the pretreated solid samples can be seen in Table 3.

### 3.1. Vine Pruning

Vine pruning (VP) consists of the remaining stalks and branches after pruning the grape trees. Different parts of grape trees have been evaluated for anaerobic digestion, such as seeds, pressings, stalks, and pomace, although there are not a lot of available works in the literature at the moment. These parts differ a lot between each other in composition as some of them might contain also soluble sugars, making the digestion less complex. The pruning that was used during this work consists of a pure lignocellulosic raw material with negligible concentration of soluble sugars and for this reason a pretreatment step might be required for the efficient production of methane. This is confirmed by the results of the digestion of the untreated VP where the obtained methane yields reached 53.8 ± 0.4 mL CH_4_/gVS. This low yield is generally expected when no pretreatment is applied to lignocellulosic biomass, due to its recalcitrance and low solubility of its complex carbohydrates (cellulose and hemicellulose).

Low yields from untreated biomass were also observed by other researchers for agricultural [14] and forest residues [15, 16]. The yield was somewhat increased for the HT sample with a methane production of 74.7 ± 11.9 mL CH_4_/gVS. Contrary to that the dilute acid pretreatment seems to have a negative effect on methane production with only 30.2 ± 2.4 mL CH_4_/gVS produced. This could be attributed to the degradation of the sugars present in cellulose and hemicellulose during the pretreatment. The most efficient pretreatment method for VP was proven to be the SE, resulting in almost doubling of the methane yield, which reached 104.1 ± 1.0 mL CH_4_/gVS.

**Figure 1:**
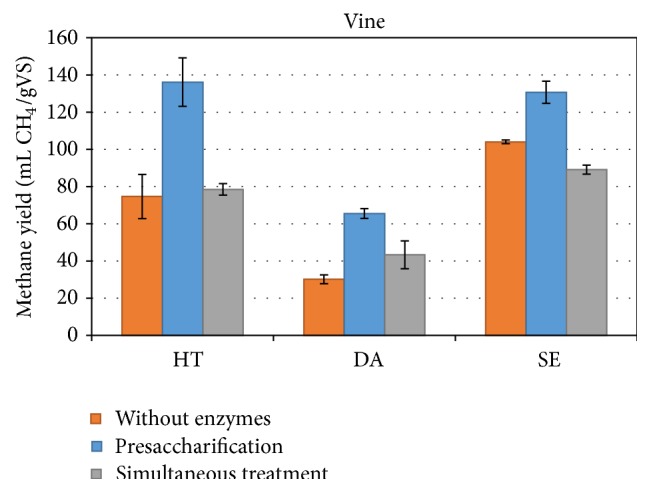
Methane yield of pretreated and enzymatically saccharified in one- and two-step process vine pruning biomass.

In the next step, the pretreated materials were further treated by enzymatic hydrolysis in an attempt to improve the obtained yields. Two different process configurations were applied: a separate and a simultaneous ones with the anaerobic digestion treatment. It can be noticed that the separate saccharification was more beneficial for all the pretreated materials. The highest methane yield was observed with the HT pretreated VP and reached 136.1 ± 13.0 mL CH_4_/gVS while the yield with the SE material was almost as high as 130.7 ± 6.0 mL CH_4_/gVS (Figure 1). On the other hand when enzymes added at the start-up of the anaerobic digestion the obtained methane yields for the HT and DA pretreated samples were lower probably due to the fact that the enzymes are not working at their optimal pH, as the pH of the anaerobic sludge was measured to be around 7.5–7.8, whereas the enzymes require slight acidic environment (pH of 5.0–5.5). The stage that the enzymes will be added in the process plays a very important role on the obtained methane yields. It was also previously shown by other authors that simultaneous treatment was less beneficial compared to the presaccharification when Jose Tall wheat grass was used [17]. In the case of the SE sample the methane production for the simultaneous enzymatic treatment is lower compared to the methane produced from the SE sample without the addition of enzymes. The positive controls with Avicel gave values of 342 mL CH_4_/gVS indicating that the sludge was active. The reduced methane production with the addition of the enzymes, therefore, hints at an inhibition of the anaerobic digestion.

As discussed above, different parts of the grape tree were used in the literature. For example, [18] used grape seeds as raw material for anaerobic digestion. They reported a yield of 173.4 mL CH_4_/gVS, although it is not easy to compare as this yield is based on both the substrate and the inoculum. Moreover, seeds have a high concentration in carbohydrates (up to 37% w/w), crude protein (up to 8.2% w/w) [19], and oils (up to 16% w/w) [20]. These compounds are easier digested compared to cellulose and hemicellulose which is the main component of the materials we used during this work. In another work [21] it was demonstrated that the pressings of the grapes can result in a methane yield of 283 mL CH_4_/gVS, whereas when the peduncles were used, the yield decreased to 180 mL CH_4_/gVS. On the other hand, when stalks were used in another work, the obtained methane yield was lower (116 mL CH_4_/gVS) compared to the one obtained during this work [22]. This underpins the fact that stalks represent one of the most recalcitrant parts of the grape tree.

### 3.2. Almond Shells

Almond shells (AS) comprise an agroindustrial waste of the almond producing industry. The most common practice to exploit AS is through burning as they offer a HHV (higher heating value) equal to 18.8 MJ/kg [23]. Research interest is focusing mostly on their gasification [24], pyrolysis [25, 26], and the production of activated carbon [27]. On the other hand, to the best of our knowledge, there is no work in the literature where AS were used as raw material for anaerobic digestion. It is, therefore, of great importance to evaluate this possibility.

When untreated material was used, the obtained yield was even lower compared to that with the untreated VP, reaching only 20.2 ± 13.0 mL CH_4_/gVS. This might be related to the different physical characteristics of the two materials, since AS are much harder and with a higher density and this might result in higher difficultly to be digested by the sludge compared to the VP. From the 3 pretreatment methods that were applied, SE were by far the most efficient as they increased the methane yields by approximately 11.5 times, resulting in a methane yield of 232.2 ± 13.0 mL CH_4_/gVS. This yield was higher than the yields obtained by using VP even after enzymatic saccharification. On the other hand, HT pretreatment did not affect the yield, whereas DA resulted in almost doubling the methane yield (38.6 ± 14.7 mL CH_4_/gVS), still well below the yield succeeded by the SE pretreatment.

**Figure 2:**
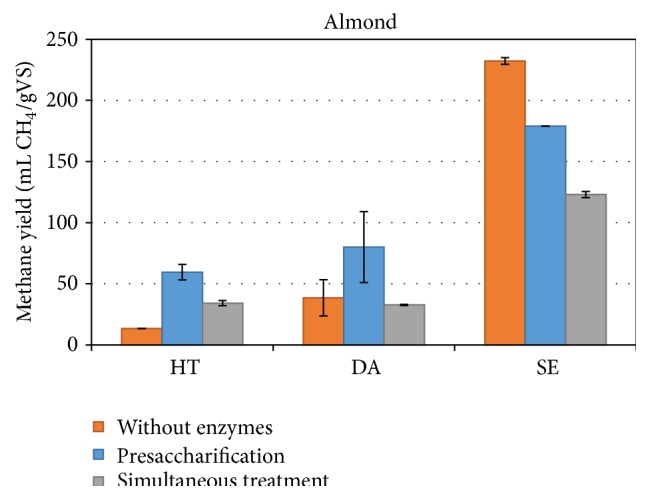
Methane yield of pretreated and enzymatically saccharified in one- and two-step process almond shells biomass.

The effect of enzymatic treatment was evaluated for AS (Figure 2). Again the presaccharification treatment was more beneficial comparing to the simultaneous enzymatic plus digestion processing for all the pretreated materials.

One interesting observation is that, with the SE pretreated AS, the application of enzymatic treatment resulted in an inhibition of the process and subsequent decrease of the methane yield. The trend was also observed in the case of the SE VP, but the reduction in methane production is greater here, for both types of enzymatic treatment. It is not very clear why the addition of enzymes had a negative effect on methane yield when SE material was used. One explanation could be that the steam explosion pretreatment was performed under high severity, leading to the degradation of xylan and formation of pseudolignin. This outer pseudolignin layer is known to irreversibly adsorb cellulolytic enzymes. The formation of additional lignin-like material in the presence of enzymes may be detrimental to methane production due to the nonproductive binding of enzymes with lignin, which could inhibit their use for methane production by the sludge. In each batch of digestion, the methane production from the organic load present in the enzyme digestion was deducted from the total to accurately represent the methane produced from the biomass only. If the enzymes were not available for digestion, however, the total methane produced could be higher.

Finally, simultaneous treatment resulted in lower yields compared to presaccharification and this is in good correlation with what was observed when VP was used.

### 3.3. Olive Pruning

Olive pruning (OP) consists of the branches that are left after pruning the olive trees. Similarly to many other agricultural wastes, OP is normally burned in order to recover energy, and the HHV is estimated close to 19.2 MJ/kg [28]. Until now, OP is evaluated as raw material for gasification [29], pyrolysis [30], ethanol production [31], and so forth, whereas as far as we know there is no report where OP was used for anaerobic digestion. When untreated OP was used the methane yield was relatively low, reaching 56.8 ± 0.3 mL CH_4_/gVS.

This value was slightly higher than the one obtained from the untreated VP. Application of pretreatment improved the methane yields with the highest yield obtained when SE was applied and reached 315.4 ± 0.0 mL CH_4_/gVS. On the other hand, the HT and DA treatments gave methane yields of 93.1 ± 1.61 mL CH_4_/gVS and 84.8 ± 0.0 mL CH_4_/gVS, respectively. Despite the fact that both HT and DA improved the methane yield, the values obtained were much lower compared to the one obtained after SE pretreatment.

**Figure 3:**
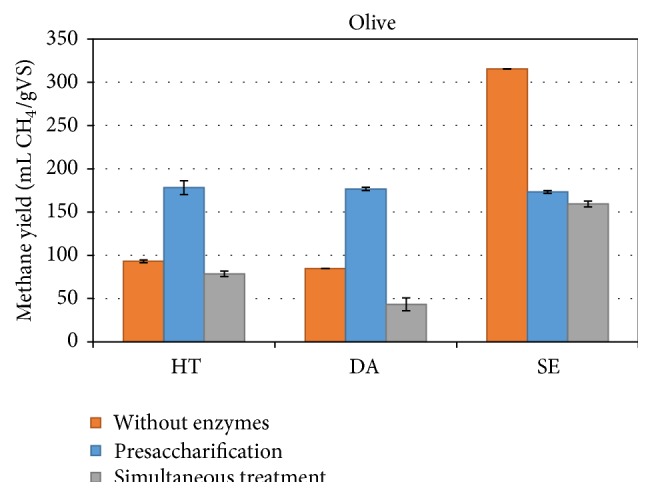
Methane yield of pretreated and enzymatically saccharified in one- and two-step process olive pruning biomass.

Finally, the effect of enzymatic treatment on the pretreated biomass was also evaluated (Figure 3).

Application of enzymes prior to digestion was once again more efficient compared to the simultaneous processing for the HT and DA material. Presaccharification almost doubled the methane yields for both HT and DA pretreated OP to 178.3 ± 8.1 mL CH_4_/gVS and 176.8 ± 1.8 mL CH_4_/gVS, respectively. The enzymatic treatment of SE pretreated samples resulted in lower yields compared to those obtained without the use of enzymes, as already seen for the VP and AS biomass. Finally, the simultaneous treatment had also a negative effect on the methane yield obtained by the DA pretreated material, whereas it did not affect the yield with the HT pretreated material. The highest yield obtained throughout this work was from the SE pretreated OP, which reached 315.4 ± 0.0 mL CH_4_/gVS.

## 4. Conclusions

In the current work three types of agricultural (olive and vine pruning) or agroindustrial (almond shells) lignocellulosic wastes were evaluated as raw material for the production of biogas. The initial untreated biomass samples gave low methane yields due to the recalcitrance of the biomass and insoluble nature of the available carbohydrates (hemicellulose and cellulose). Three methods were used to improve the digestibility of biogas, namely, hydrothermal, dilute acid, and steam explosion pretreatment. Depending on the type of biomass hydrothermal and dilute acid pretreatment provided from little to moderate improvement to the methane yield. Steam explosion proved to be the more favorable pretreatment method for all three biomass types, but particularly for almond shells and olive pruning where the highest methane yields of 232.2 ± 13.0 mL CH_4_/gVS and 315.4 ± 0.0 mL CH_4_/gVS, respectively, were achieved.

Presaccharification of the pretreated biomass samples provided controversial results. It improved methane yields for hydrothermal and dilute acid pretreatment methods, where the initial positive effect of the pretreatment was moderate. In the case of steam explosion, however, where a high positive effect for the digestion was achieved, the introduction of the extra enzymatic hydrolysis step provided a negative effect for all three types of biomass. The addition of enzymes directly to the digestion vessel provided a smaller yield compared to the prehydrolysis, due to the nonoptimal for the enzymes pH of the digestion medium.

In conclusion almond shells and olive pruning pretreated with steam explosion provided the best methane yields compared to all the other process parameters evaluated in the current work. A careful optimization of the pretreatment and the enzymatic hydrolysis parameters would enable us to determine if the methane yields of all three biomass types can be further improved.

## Figures and Tables

**Table 1:** Volatile solids (VS) and total solids (TS) content of initial untreated biomass samples.

Sample	Total solids (wt. %)	Volatile solids (wt. %)
Vine pruning	95.07	91.97
Olive pruning	91.63	89.02
Almond shells	93.06	91.95
Inoculum	1.53	0.97

**Table 2:** Experimental conditions of hydrothermal (HT), dilute acid (DA), and steam explosion (SE) pretreatment.

Type of pretreatment	Solids (w/v)	*T* (°C)	*t* (min)	Catalyst (w/w)
Hydrothermal (HT)	10%	200	7	- no -
Dilute acid (DA)	10%	170	13	1% H_2_SO_4_
Steam explosion (SE)	37.5%	195	10	1% H_2_SO_4_

**Table 3:** Volatile solids (VS) and total solids (TS) of biomass samples before and after pretreatment with hydrothermal (HT), dilute acid (DA), and steam explosion (SE) methods.

Sample	Untreated		HT		DA		SE	
	TS	VS	TS	VS	TS	VS	TS	VS
Vine pruning	95.07	91.97	98.31	96.06	98.17	98.02	93.64	90.63
Olive pruning	91.63	89.02	99.78	97.56	100.00	100.00	88.35	85.72
Almond shells	93.06	91.95	100.00	100.00	98.69	98.69	96.45	95.70
